# Hypoxic dental pulp stem cells-released succinate promotes osteoclastogenesis and root resorption

**DOI:** 10.7150/ijms.94972

**Published:** 2024-04-29

**Authors:** Andi Yang, Jinmeng Wang, Zhiyu Yang, Lulu Wang, Houxuan Li, Lang Lei

**Affiliations:** 1 Nanjing Stomatological Hospital, Affiliated Hospital of Medical School of Nanjing University, Nanjing, China.; 2 Central Laboratory of Stomatology, Nanjing Stomatological Hospital, Affiliated Hospital of Medical School of Nanjing University, Nanjing, China.

**Keywords:** Dental pulp stem cells, root resorption, hypoxia, succinate

## Abstract

**Introduction:** Clinical studies have shown that endodontically-treated nonvital teeth exhibit less root resorption during orthodontic tooth movement. The purpose of this study was to explore whether hypoxic dental pulp stem cells (DPSCs) can promote osteoclastogenesis in orthodontically induced inflammatory root resorption (OIIRR).

**Methods:** Succinate in the supernatant of DPSCs under normal and hypoxic conditions was measured by a succinic acid assay kit. The culture supernatant of hypoxia-treated DPSCs was used as conditioned medium (Hypo-CM). Bone marrow-derived macrophages (BMDMs) from succinate receptor 1 (SUCNR1)-knockout or wild-type mice were cultured with conditioned medium (CM), exogenous succinate or a specific inhibitor of SUCNR1 (4c). Tartrate-resistant acid phosphatase (TRAP) staining, Transwell assays, qPCR, Western blotting, and resorption assays were used to evaluate osteoclastogenesis-related changes.

**Results:** The concentration of succinate reached a maximal concentration at 6 h in the supernatant of hypoxia-treated DPSCs. Hypo-CM-treated macrophages were polarized to M1 proinflammatory macrophages. Hypo-CM treatment significantly increased the formation and differentiation of osteoclasts and increased the expression of osteoclastogenesis-related genes, and this effect was inhibited by the specific succinate inhibitor 4c. Succinate promoted chemotaxis and polarization of M1-type macrophages with increased expression of osteoclast generation-related genes. SUCNR1 knockout decreased macrophage migration, M1 macrophage polarization, differentiation and maturation of osteoclasts, as shown by TRAP and NFATc1 expression and cementum resorption.

**Conclusions:** Hypoxic DPSC-derived succinate may promote osteoclast differentiation and root resorption. The regulation of the succinate-SUCNR1 axis may contribute to the reduction in the OIIRR.

## Introduction

Dental pulp stem cells (DPSCs) reside in the solid house of the pulp chamber and root canal. Mesenchymal stem cells possess high self-renewal potential and regeneration capacity and form dentin/pulp-like complexes during tooth development. DPSCs are nourished by a rich blood supply from arterial branches extending from the apical foramina to maintain pulp homeostasis 1.

Reduced pulp blood flow (PBF) after the application of orthodontic force, such as aligning, levelling and space closing, has long been recognized among orthodontic and endodontic scholars 2-4. The reduction in PBF is most prominent during intrusion of the incisor tooth 5-7. DPSCs may survive the hypoxic niche during orthodontic tooth movement (OTM), as evidenced by the rare incidence of pulp necrosis in orthodontic clinics 8,9. It has been demonstrated that intrusion is a key risk factor for orthodontically induced inflammatory root resorption (OIIRR), especially for teeth with malformed roots 10,11. The correlation between the reduced PBF and OIIRR has never been explored.

Although OIIRR is considered to be a sterile inflammatory process closely related to osteoclast activity in the periodontium, the mechanism underlying the root resorption process is largely unknown, currently 12. Several specific molecular pathways have been investigated to explore the molecular mechanism that orchestrates root resorption, such as receptor activator of nuclear factor kappa-B ligand (RANKL)/RANK/osteoprotegerin and adenosine triphosphate (ATP)-purinergic receptor P2X7 (P2X7R)-interleukin-1 pathways, to activate osteoclastic cell fusion and activation. In addition, at the cellular level, polarization of macrophages has also been implicated in the process of OIIRR; more specifically, the balance between CD68^+^, iNOS^+^ proinflammatory M1-like macrophages and CD68^+^, CD163^+^ anti-inflammatory M2-like macrophages impacts the process of OIIRR 13. Although pathways linked to root resorption have been extensively discussed, the signal that triggers root resorption have seldom been investigated.

Living cells rely mostly on glucose metabolism to provide energy and sustain numerous biological processes. When the oxygen supply is sufficient, cells mostly depend on the tricarboxylic acid cycle to generate substrates for the mitochondrial electron transport chain and oxidative phosphorylation to produce adenosine triphosphate 14. When the oxygen supply is restricted, living cells may rewire their energy from oxidative phosphorylation to anaerobic glycolysis, providing fast energy production and sufficient substrates, such as nicotinamide adenine dinucleotide + hydrogen (NADH) and nicotinamide adenine dinucleotide phosphate (NADPH), for glycogen, lipid, nucleotide and protein synthesis 15.

The metabolic reprogramming from oxidative phosphorylation to glycolysis is accompanied by the shutdown of the TCA cycle (tricarboxylic acid cycle) with dampened activity of succinate dehydrogenase (SDH), a key TCA cycle enzyme and an integral component of the mitochondrial respiratory chain complex II 16. Such disruption of the TCA cycle at SDH may lead to the accumulation of succinate in the mitochondria, which may be transported into the cytosol and the extracellular space. Neighboring cells can sense succinate via succinate receptor 1 (SUCNR1), leading to a cascade of biological events in cells 17. Therefore, succinate may play an important role in transmitting metabolic signals in mitochondria.

The role of dental pulp in OIIRR has been long recognized in the dental society, since nonvital teeth after root canal therapy showed reduced OIIRR when compared to its vital counterpart 18,19; however, the role of pulp tissues in the OIIRR has not been fully uncovered. OIIRR may be rather different from inflammatory root resorption in severely traumatized teeth. The physical damage, bacterial involvement and necrosis of periodontal ligaments and pulp tissues in traumatized teeth may trigger an intense immuno-inflammatory response, resulting in both external replacement root resorption and inflammatory root resorption 20. Orthodontic treatment can affect the cellular metabolism of the dental pulp 21, shown by the increased activity of aspartate aminotransferase as a result of hypoxia following circulatory disruptions in the pulp tissues 22. Based on the nature of inflammation and metabolism, we inferred that hypoxic DPSCs in the reduced PBF state may release metabolic signals to induce root resorption, increase apical restriction and restore the blood supply. The purpose of the present study was to explore whether hypoxic DPSCs release succinate and its role in triggering osteoclastogenesis and root resorption.

## Materials and Methods

### Animals

SUCNR1^-/-^ (SUCNR1 knockout) and wild-type (WT) mice on the C57BL6/J background were generated and identified by GemPharmatech (Nanjing, China). The room temperature and humidity were controlled at 22 ± 1 °C and 40-60%, respectively. Mice were housed under a 12-hour light/dark cycle and fed tap water and soft food. Animal care and experimental protocols were approved by Ethics Committee of Nanjing Stomatological Hospital, Affiliated Hospital of Medical School of Nanjing University ((No. IACUC-D2303123& No. NJSH-2023NL-27).

### Cell culture and collection of conditioned medium

Human DPSCs were isolated from the third molars of participants aged 18~20 years, and written informed consent was obtained from the patients. Cells were seeded in a T-25 tissue culture flask supplemented with 10% fetal bovine serum (FBS, Gibco, USA) and incubated under normal oxygen conditions (21% O_2_) or hypoxic conditions (1% O_2_) for the indicated times (6 h) 23. The two cell supernatants were collected, designated Norm-CM (conditional medium under normal oxygen conditions) and Hypo-CM (conditional medium under hypoxic oxygen conditions) and used to conditionally culture macrophages or osteoclasts.

### Bone marrow-derived macrophage and osteoclast culture

Bone marrow-derived macrophages from mice (mBMDMs) were isolated from SUCNR1^-/-^ or WT mice and differentiated in RPMI 1640 medium (Gibco, USA) supplemented with 10% FBS and 50 ng/ml macrophage colony-stimulating factor (M-CSF) for six days under normal oxygen conditions (21% O_2_) as previously described 24. BMDMs were seeded in 96-well plates, cementum pieces and Osteo assay surface plates (Corning, USA) and cultured with 30 ng/ml M-CSF and 50 ng/ml RANKL for 7 days to induce differentiation into osteoclasts. Fresh cytokines were added every other day. After the osteoclasts matured, the experiments were performed.

### Tartrate-resistant acid phosphatase (TRAP)

To perform TRAP staining, the cell cultures were fixed in precooled 4% paraformaldehyde for 10 min and then incubated with *TRAP stain solution* (Wako, Japan) for 15 min at 37 °C, followed by dilution with distilled deionized water. Then, 250 μL of *nuclear stain solution* (Wako, Japan) was added to the wells, and the cells were stained for 10 min at the room temperature. TRAP-positive cells were identified and counted as osteoclasts using light microscopy as described previously 25.

### Succinate measurement, exogenous succinate treatment and succinate receptor antagonist treatment

The amount of succinate in the culture supernatants was measured by a succinate colorimetric assay kit (Megazyme, Ireland) according to the manufacturer's instructions 16. Exogenous succinate (Sigma‒Aldrich, USA) was added to the medium to stimulate osteoclastogenesis at the designated concentrations (1, 5, or 25 mM). The SUCNR1-antagonist 4c was synthesized by Wuhe BioTech Co., China, and 4c (10 μM) was added to the culture medium 2 h before stimulation.

### Fixation and staining of cementum pieces

Healthy third molars were processed from 2 mm × 2 mm × 2 mm cementum pieces at 2 mm under the cementum-enamel junction 26. Cementum slices were laid flat on a 96-well plate. mBMDMs were seeded at 200,000 per well in 96-well plates. The cells were incubated in media supplemented with 30 ng/ml M-CSF and 50 ng/ml RANKL for 5 days. BMDMs were allowed to settle on the cementum slices 27. Then, the cells were treated with CM or exogenous succinate. At the end of the culture period, the cementum pieces were washed in PBS at 37 °C, fixed in 4% paraformaldehyde, and stained for 10 min in hematoxylin (Beyotime, China). Excess stain was removed by washing in distilled water, and the sample was air-dried. To quantify the resorption areas, five areas of the cementum slices were randomly selected, and the areas of the stained resorption lacunae were observed under an inverted optic microscope (Nikon, Japan).

### Flow cytometry

After treatment with CM, succinate or 4c for 48 h, the macrophages were washed, trypsinized, and resuspended in PBS containing 1% FBS. Next, the cells were incubated with surface markers (FITC-conjugated anti-mouse CD11b, FITC-conjugated rat IgG1, κ isotype control, PE-conjugated rat IgG2b, κ isotype control and PE-conjugated rat IgG1, κ isotype control; all from Biolgend, USA) for 10 min. Cells incubated with FITC-conjugated anti-mouse CD11b were divided into two tubes and stained with PE-conjugated anti-mouse CD206 or PE-conjugated anti-mouse CD86. After 15 min, the cells were analyzed by flow cytometry (BD Biosciences, USA) 28. The polarization of mBMDMs to the M1 phenotype (M1-like macrophages) was further verified with CD11b^+^ and CD86^+^ macrophages. CD11b^+^ and CD206^+^ macrophages were M2-like macrophages. The M1/M2 macrophage ratio at 48 h was analyzed to determine the trend in the polarization of mBMDMs toward the M1 or M2 phenotype.

### Resorption assays

mBMDMs isolated from SUCNR1^-/-^ or WT mice were seeded at 800,000 cells/well in 24-well Osteo assay surface plates (Corning, USA) supplemented with 30 ng/ml M-CSF, 50 ng/ml RANKL and exogenous succinate or 4c. We utilized 10% bleach to wash away the cells after OC culture, and the cells were air-dried. Images of the resorption pits on the Osteo assay surface plates were acquired under an inverted light microscope. We detected and calculated the pit formation ratio (pit area versus total area) based on the images obtained 29.

### Real-time PCR

Total RNA was extracted with total RNA extraction reagent (Tiangen, China) and reverse transcribed with a cDNA synthesis kit (Vazyme, China). The sequences of the primers used for quantitative real-time PCR (qPCR) were obtained from PrimerBank and synthesized by GenScript (GenScript, China). The relative quantification was calculated by the comparative 2^-∆∆Ct^ method. We used a CFX384 Touch Real-Time PCR Detection System (Bio-Rad, USA). The primers used were as follows in Table 1.

### Transwell tests

mBMDMs (1 × 10^5^/well) isolated from SUCNR1^-/-^ or WT mice were loaded into the upper chamber in 150 μL of RPMI 1640 medium supplemented with 5% FBS. Normal medium containing succinate or 4c and conditioned medium were added to the lower chambers of Transwell inserts with a pore size of 8 μm (Corning, USA). After incubation at 37 °C with 5% CO_2_ for 5 days, the mBMDMs were rinsed with PBS, fixed in 4% paraformaldehyde for 15 min and stained with 600 μL of crystal violet staining solution (Beyotime, China) for 10 min. Cells that had migrated to the lower surface of the membrane were counted in five randomly selected fields under an inverted light microscope as described previously 30.

### Western blotting

Western blotting was conducted as we reported previously 16. Briefly, the concentration of total proteins in the lysed cells was detected utilizing a Nanodrop (Thermo Fisher, USA). Proteins were subjected to 4-12% SDS‒PAGE and transferred to a PVDF membrane. The membranes were blocked with 5% BSA and incubated with anti-SUCNR1, anti-TRAP (Proteintech, China), anti-MMP9 (CST, Germany), and anti-GAPDH (Servicebio, China) secondary antibodies. The signals were detected using Tanon-5200.

### Quantification and statistical analysis

The analysis was performed using Graph Pad Prism 8.0 software. All results are presented as the mean ± standard deviation (SD). We used analysis of variance when the study subjects included more than two groups, followed by the Bonferroni t test. Student's t test was used to analyze differences between two groups. Values of p < 0.05 were considered to indicate statistical significance. All *in vitro* experiments were independently performed as indicated.

## Results

### Hypoxic DPSCs released succinate and promoted the polarization of mBMDMs with increased destruction of the cementum

**Figure 1:**
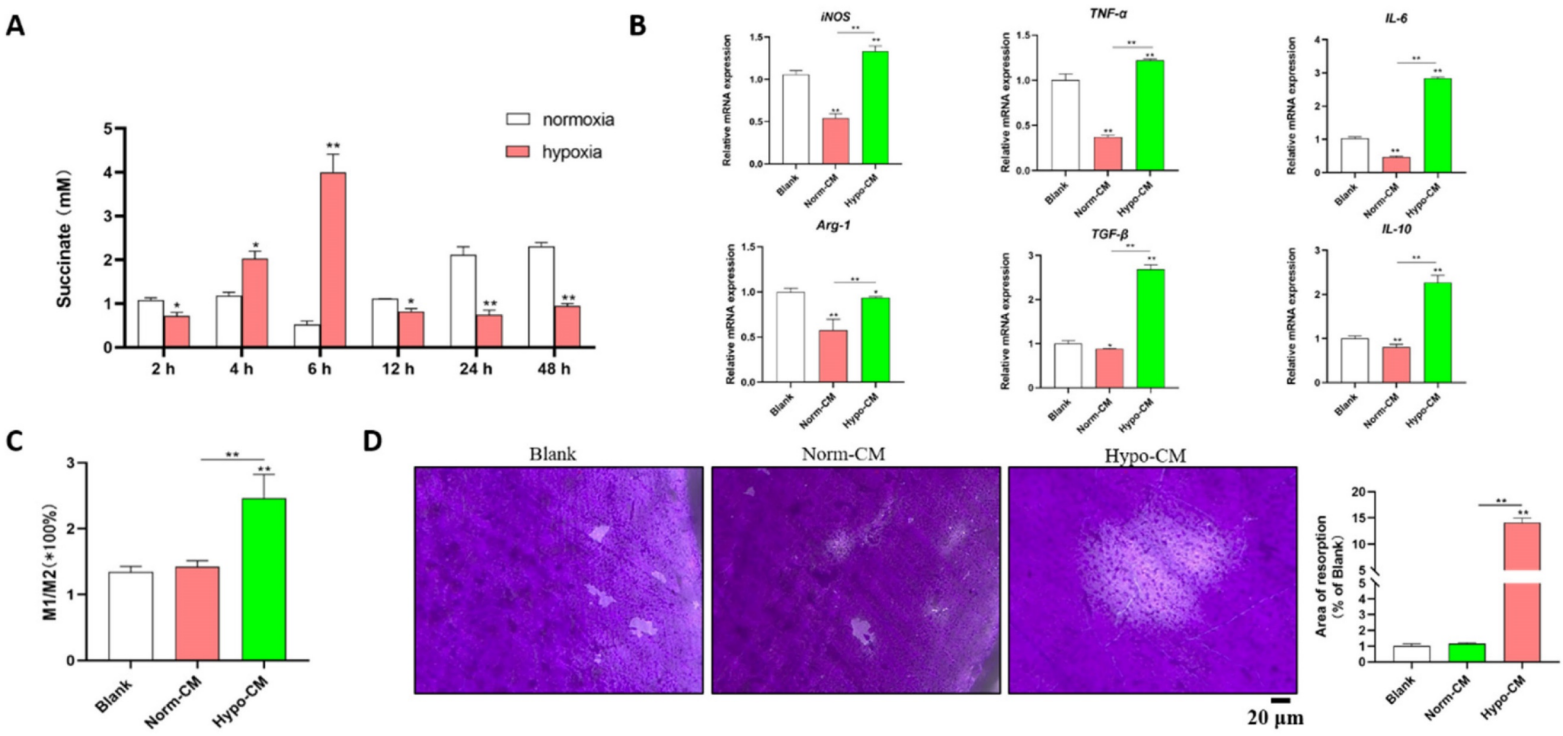
** Succinate from hypoxic DPSCs promoted M1 macrophage polarization and cementum resorption.** (A) Succinate in the supernatant of normoxia- and hypoxia-cultured DPSCs was determined by a colorimetric assay. (B) mBMDMs from WT mice were cultured with normal CM or Hypo-CM supplemented with 30 ng/ml M-CSF and 50 ng/ml RANKL for 7 days, and the transcription of polarization-related genes at 2 h was measured by real-time PCR. (C) The M1/M2 macrophage ratio at 48 h was analyzed by flow cytometry. (D) Cementum resorption by osteoclasts was evaluated after 7 days. *p < 0.05, **p < 0.001.

Under hypoxic conditions, the levels of succinate in the culture supernatants of DPSCs increased after 2 h, peaked at 6 h (approximately 4 mM), decreased at 12 h and remained stable until 48 h (Figure 1A). The supernatants of DPSCs cultured under hypoxic and normal conditions for 6 h were collected as conditioned media for mBMDMs, which were designated Hypo-CM and Norm-CM, respectively. Compared with normoxia, Norm-CM reduced iNOS, TNF-α, IL-6, Arg-1 and IL-10 gene transcription at 24 h after treatment, while Hypo-CM promoted iNOS, TNF-α, IL-6, Arg-1, TGF-β and IL-10 gene transcription. A more than 1-fold increase in the M1/M2 ratio was observed via flow cytometry in Hypo-CM-treated DPSCs (Figure 1C). Moreover, we seeded BMDMs onto the cementum plates, and the area of resorbed cementum was increased in the Hypo-CM-treated osteoclasts after 7 days when compared with the blank or Norm-CM groups (Figure 1D).

### 4c inhibited the ability of succinate to promote osteoclast formation and differentiation

**Figure 2:**
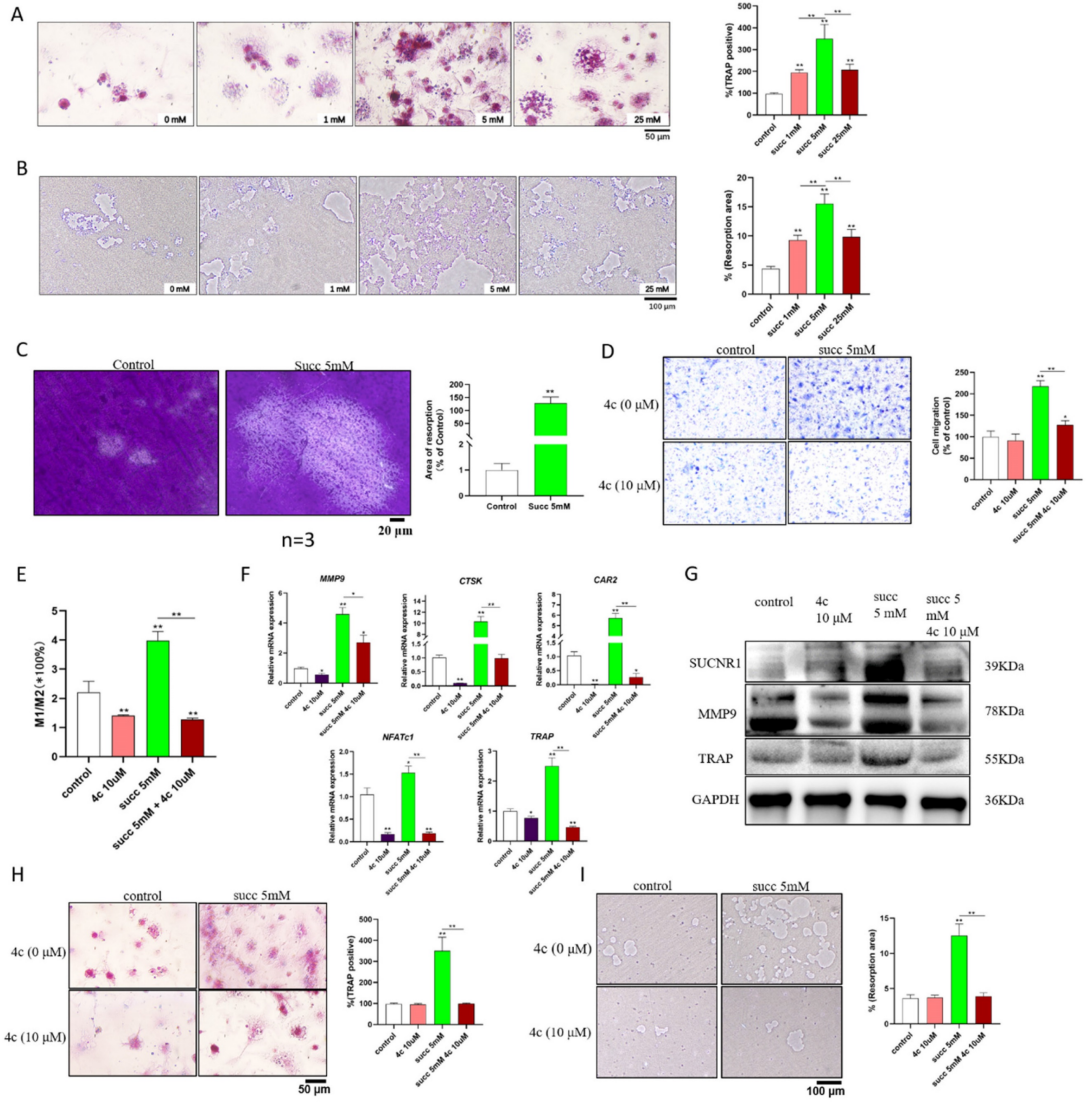
** Exogenous succinate promoted osteoclast formation and cementum resorption.** BMDMs from WT mice were cultured with 30 ng/ml M-CSF and 50 ng/mL RANKL to induce osteoclast formation, and 1, 5, or 25 mM succinate or 10 μM 4c was added to the culture medium. (A) TRAP staining was used to measure osteoclast formation. (B) Resorption pits were detected on the Osteo assay surface plates. (C) Cementum resorption was observed under a microscope. (D) The migration of mBMDMs was detected by a Transwell assay after 5 days of treatment with succinate in the lower chamber. (E) The M1/M2 macrophage polarization ratio was analyzed by flow cytometry after 48 h. (F) Polarization-related mRNA transcription was determined by qPCR at 2 h. (G) The protein expression of SUCNR1, MMP9 and TRAP was tested by western blotting at 24 h. (H&I) Osteoclast formation was measured by TRAP staining, and resorption pits on the Osteo assay surface plates were observed on Day 7. *p < 0.05, **p < 0.001.

To further address the role of succinate in promoting osteoclastogenesis and cementum resorption, we utilized exogenous succinate to stimulate BMDMs. According to the results shown in Figure 1A, the greatest amount of succinate that was spontaneously secreted by hypoxic DPSCs was approximately 4 mM. Therefore, we used 0 mM, 1 mM, 5 mM and 25 mM exogenous succinate to stimulate BMDMs. The formation of osteoclasts and resorption pits peaked in 5 mM succinate-treated BMDMs and osteoclasts (Figure 2A&B); in addition, succinate promoted cementum resorption (Figure 2C). Next, we explored whether 4c, a specific antagonist of SUCNR1, could reduce osteoclastogenesis. A 2-fold increase in the migration of monocytes was observed in the medium supplemented with succinate in the Transwell assays, while 4c (10 μM) reduced the migration of monocytes; in addition, 4c decreased M1 macrophage polarization (Figure 2D&E); moreover, pretreatment with 4c reduced the transcription of osteoclastogenesis-related genes, including matrix metalloproteinase-9 (MMP9), cathepsin K (CTSK), carbonic anhydrase 2 (CAR2), nuclear factor of activated T cells 1 (NFATc1), and tartrate-resistant acid phosphatase (TRAP), and reduced the protein levels of SUCNR1, MMP-9 and TRAP (Figure 2F&G); furthermore, pretreatment with 4c reduced the number of TRAP-positive cells and the area of resorption pits in succinate-treated BMDMs (Figure 2H&I).

### 4c inhibited the ability of Hypo-CM to promote osteoclast formation

**Figure 3:**
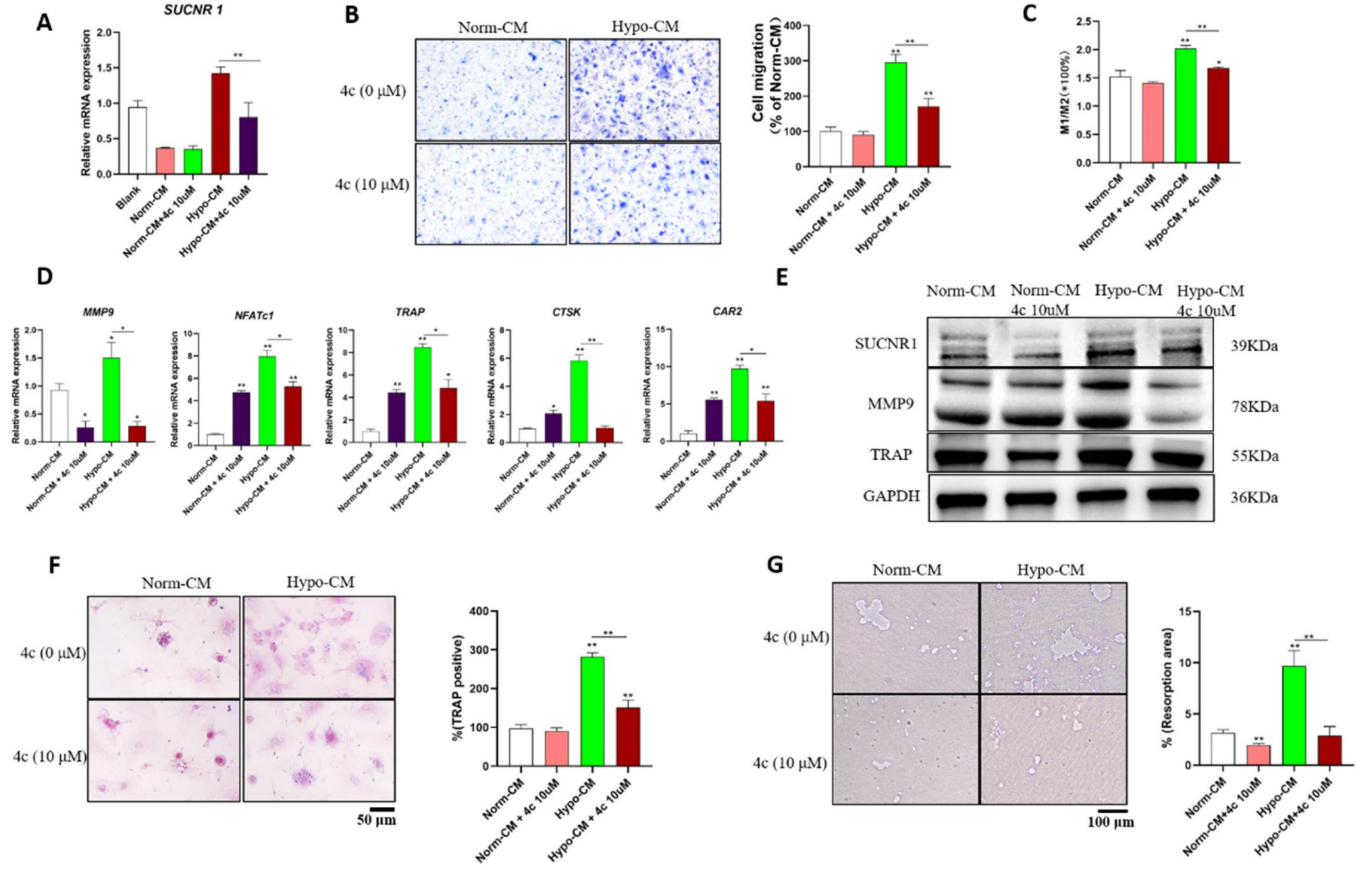
** Hypo-CM stimulated osteoclastogenesis via SUCNR1.** BMDMs from WT mice were cultured with 30 ng/ml M-CSF and 50 ng/mL RANKL to induce osteoclast formation, and Hypo-CM or Norm-CM was added to the culture medium. (A) The gene transcription of SUCNR1 in BMDMs was tested at 2 h. (B) The migration of mBMDMs was detected by Transwell assay after 5 days of culture in conditioned medium in the lower chamber. (C) Macrophage polarization was determined by flow cytometry at 48 h. (D) Polarization-related mRNA transcription was determined by qPCR at 2 h. (E&F) Gene transcription and protein expression were tested by real-time PCR and western blotting, respectively. (G) Resorption pits on the Osteo assay surface plates were observed on Day 7. *p < 0.05, **p < 0.001.

To further characterize whether Hypo-CM promoted osteoclast formation in a succinate-dependent manner, we further explored whether 4c could reduce Hypo-CM-promoted osteoclast formation. Hypo-CM promoted SUCNR1 transcription, while 4c reduced SUCNR1 transcription (Figure 3A). The SUCNR1 antagonist 4c reduced the migration of Hypo-CM-treated macrophages according to Transwell assays and decreased M1 macrophage polarization according to flow cytometry (Figure 3B&C). Pretreatment with 4c reduced the transcription of MMP9, NFATc1, TRAP, CTSK and CAR2 and decreased the protein expression of MMP9 and TRAP in Hypo-CM-treated BMDMs (Figure 3D&E). Moreover, the number of TRAP-positive cells and the area of resorption pits were decreased by 4c pretreatment in Hypo-CM-treated BMDMs (Figure 3F&G).

### SUCNR1 knockout inhibited Hypo-CM-promoted osteoclast formation

**Figure 4:**
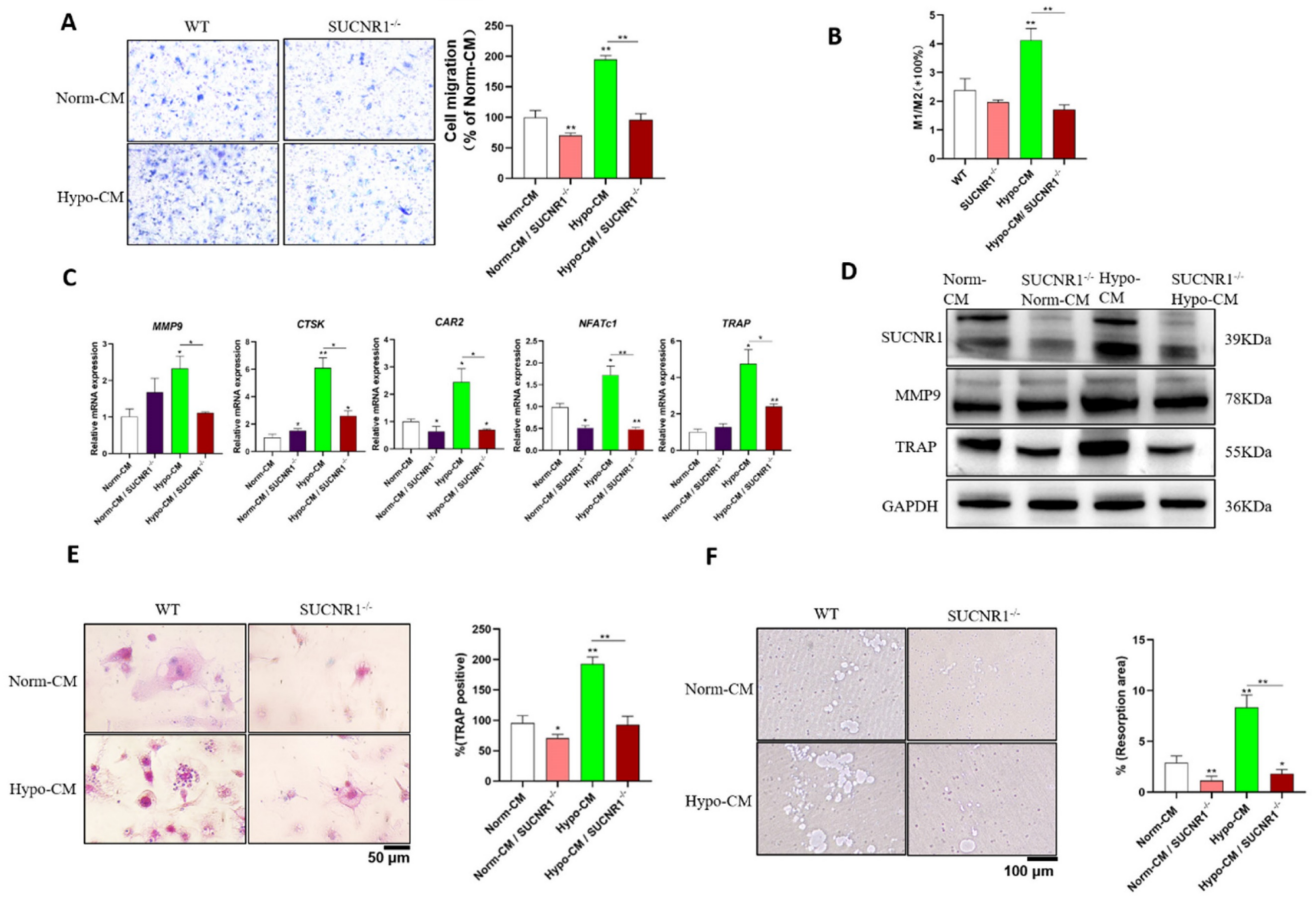
** SUCNR1 gene knockout decreased macrophages, migration, polarization and osteoclast differentiation in response to Hypo-CM treatment.** mBMDMs from SUCNR1^-/-^ or WT mice were cultured with M-CSF and RANKL to induce osteoclast formation, and Hypo-CM or Norm-CM was added to the culture medium. (A) Cell migration was quantified with Hypo-CM or Norm-CM in the lower chamber for 5 days. (B) The M1/M2 ratio was analyzed by flow cytometry at 48 h. (C) Gene transcription was determined by quantitative real-time PCR at 2 h. (D) The protein expression of SUCNR1, MMP9 and TRAP was tested by western blotting at 48 h. (E) TRAP staining of osteoclasts was performed on Day 7. (F) Resorption pits on Osteo assay plates were observed after 7 days. *p < 0.05, **p < 0.001.

To further characterize the mechanism by which Hypo-CM from DPSCs promotes osteoclastogenesis and root resorption, we cultured BMDMs from SUCNR1 knockout mice. Reduced migration was observed in Hypo-CM-treated BMDMs from SUCNR1^-/-^ mice (Figure 4A); similarly, decreased polarization of M1 macrophages was found in BMDMs from SUCNR1^-/-^ mice after Hypo-CM treatment (Figure 4B). In SUCNR1^-/-^ osteoclasts treated with Hypo-CM, the transcription of MMP9, CTSK, CAR2, NFATc1, and TRAP decreased, while the protein expression of MMP9 and TRAP decreased in BMDMs from SUCNR1^-/-^ mice (Figure 4C&D). Moreover, fewer TRAP-positive cells and fewer resorption pits were found in the SUCNR1^-/-^ BMDMs after Hypo-CM treatment (Figure 4E&F).

## Discussion

The OIIRR is a critical sequela of orthodontic tooth movement. M1 macrophage polarization and decreased cementoblast mineralization have been implicated as potential mechanisms of OIIRR 31. However, the signaling pathways involved in monocyte chemotaxis, macrophage polarization and osteoclast formation in OIIRR have not been fully characterized. During OTM, especially intrusion, blood vessels at the root apex are constricted or occluded, leading to reduced pulpal blood flow and a hypoxic state in the pulp tissues 32.

Resorption of the root apex may relieve the strain in the apical area and recover the blood supply to the pulp tissues. Therefore, metabolic signals from DPSCs might play a role in triggering OIIRR during OTM. Since most orthodontic force-induced root resorption is initiated from the apex and a reduced OIIRR is observed in endodontically treated teeth 18, our present study demonstrated for the first time that hypoxic DPSCs generated succinate, which triggers osteoclastogenesis from mBMDMs and root resorption; therefore, the OIIRR may be a defense mechanism of pulp tissues to fulfill oxygen needs during OTM.

DPSCs dynamically alter their cellular metabolic activities to adapt to individual conditions. These cells undergo a metabolic shift to enhanced oxidative phosphorylation when cultured in osteogenic medium 33. The metabolic status of pulp tissue during OTM has seldom been studied in the disciplines of endodontics and orthodontics. Pulp tissue may metabolically adapt to rapid palatal expansion with increased aspartate aminotransferase (AST) activity 34. In addition, enhanced AST activity is observed in human premolars after 2 weeks of orthodontic intrusion 9. DPSCs can survive long-term hypoxia 35; however, changes in glucose metabolism have never been addressed. In our present study, hypoxia increased succinate accumulation in the cytosol, and such accumulated succinate can be released and detected in the extracellular space.

Disruption of the balance between RANKL and OPG expression in periodontal tissue has been proposed as an important mechanism for OIIRR, and heavy orthodontic forces promote osteoclastogenesis and enhance external root resorption by upregulating RANKL expression in rat periodontal tissues 36. In addition, the OIIRR has been also ascribed to reduced mineralization capacity 37 and apoptosis of cementoblast 38, as well as enhanced M1 macrophage polarization 39-41. The abovementioned theory cannot fully explain the phenomenon of reduced OIIRR in endodontically treated teeth (ETT) 42. For example, the surface area reduction after OTM was 13.01 mm^2^ and 19.95 mm^2^ in ETT and vital pulp teeth, respectively 43, which suggests vital pulp tissues may release signals to trigger OIIRR. Our present study indicated that succinate, a metabolite signal released from hypoxic DPSCs during orthodontic tooth movement, promoted macrophage polarization and osteoclastogenesis. However, whether the succinate from hypoxic DPSCs would affect mineralization capacity and cell survival of cementoblasts needs to be further explored.

By transmitting the metabolic signal of dampened oxidative phosphorylation in the mitochondria, succinate transmits a proinflammatory signal and plays an important role in many critical pathophysiologic processes, including innate immunity, inflammation, tissue repair, and oncogenes. For example, *Porphyromonas gingivalis*, a keystone periodontal pathogen, triggers succinate accumulation and inflammatory responses in periodontal ligament cells 44. In addition, a SUCNR1 antagonist reduced periodontal bone loss in mice *in vivo* 17. Coupling of succinate with SUCNR1 promotes NF-κB activation in osteoclasts 45, and it also activates Ca^2+^-dependent mitogen-activated protein kinase-extracellular signal-regulated kinases 1/2 (MAPK-ERK1/2) pathways, leading to increased nitric oxide generation and prostaglandin E2 production 46. Therefore, succinate released from hypoxic DPSCs may trigger profound inflammatory biological events in periodontal tissues, leading to macrophage polarization, osteoclastogenesis and root resorption.

Although we focused on succinate and SUCNR1 in the present study, the regulatory roles of other signals should also be further investigated. For example, hypoxic DPSCs release exosomes to enhance angiogenesis 47. Small extracellular vesicles released from hypoxic DPSCs inhibit inflammatory osteolysis by reducing macrophage polarization and osteoclastogenesis 48. This phenomenon indicated that the role of hypoxic DPSCs in macrophage polarization and osteoclastogenesis might be complicated. Indeed, a fluctuation in the succinate concentration in the culture medium of hypoxia-treated DPSCs was observed in our present study. In addition, we observed that the SUCNR1 antagonist reduced osteoclastogenesis and root resorption, which may also indicate that the succinate-SUCNR1 axis may participate in OTM and that targeting SUCNR1 may reduce not only root resorption but also OTM.

There were some study limitations as follows. Accurately, it was not easy to distinguish cementum or dentin of 2mm x 2mm x 2mm of tooth structure in our experiment. Root resorption would be observed in both cementum and dentin by Micro-CT 49; therefore, root resorption in our cementum plates may contain both dentin and cementum components. Furthermore, resorption in cementum and dentin may be different and need to be further explored. Moreover, animal experiments were needed to further explore the role of metabolic changes of pulp tissues in triggering root resorption.

In conclusion, our present study indicated that hypoxia triggered succinate accumulation in the cytosol and the culture medium, releasing an inflammatory signal for macrophage polarization, osteogenesis and root resorption, suggesting an important role of DPSCs in initiating OIIRR during OTM.

## Figures and Tables

**Table 1:** Sequences of the primers.

Primers	Fr	Rv
iNOS	5'-CAGCTGGGCTGTACAAACCTT-3'	5'-CATTGGAAGTGAAGCGTTTCG-3'
IL-10	5'-ACTCTTCACCTGCTCCACTG-3'	5'-GCTATGCTGCCTGCTCTTAC-3'
TNF-α	5'-TCTTCTCATTCCTGCTTGTGG-3'	5'-GAGGCCATTTGGGAACTTCT-3'
Arg-1	5'- CCAGATGTACCAGGATTCTC -3'	5'-AGCAGGTAGCTGAAGGTCTC-3'
TGF-β	5'-CTTCAGCCTCCACAGAGAAGAACT-3'	5'-TGTGTCCAGGCTCCAAATATAG-3'
IL-6	5'-TCCACGATTTCCCAGAGAAC-3'	5'-TCCACGATTTCCCAGAGAAC-3'
ACTIN	5'-GGAGATTACTGCCCTGGCTCCTA-3'	5'-GACTATCGACTCCTGCTTGCTG -3'
